# The Relationship between Executive Functions, Working Memory, and Intelligence in Kindergarten Children

**DOI:** 10.3390/jintelligence11040064

**Published:** 2023-03-29

**Authors:** Ebru Ger, Claudia M. Roebers

**Affiliations:** Institute of Psychology, University of Bern, 3012 Bern, Switzerland

**Keywords:** hearts and flowers, position span task, RIAS, backward color recall task, cognitive control, post-error slowing

## Abstract

Executive functions (EF), working memory (WM), and intelligence are closely associated, but distinct constructs. What underlies the associations between these constructs, especially in childhood, is not well understood. In this pre-registered study, along with the traditional aggregate accuracy and RT-based measures of EF, we investigated post-error slowing (PES) in EF as a manifestation of metacognitive processes (i.e., monitoring and cognitive control) in relation to WM and intelligence. Thereby, we aimed to elucidate whether these metacognitive processes may be one underlying component to explain the associations between these constructs. We tested kindergarten children (*M*_age_ = 6.4 years, *SD*_age_ = 0.3) in an EF, WM (verbal and visuospatial), and fluid (non-verbal) intelligence task. We found significant associations of mainly the inhibition component of EF with fluid intelligence and verbal WM, and between verbal WM and intelligence. No significant associations emerged between the PES in EF and intelligence or WM. These results suggest that in the kindergarten age, inhibition rather than monitoring and cognitive control might be the underlying component that explains the associations between EF, WM, and intelligence.

## 1. Executive Functions (EF), Working Memory (WM), and Intelligence

“Jacob is a very intelligent student. He was able to read fluently before starting first grade, and ever since, he scores high on math tests, is the best in science, and engages in computer programming in his leisure time. Yet, his teacher complains about his classroom behavior. He often shouts in the classroom, runs into conflicts with his classmates, especially during sports lessons, and surprises his teacher every so often with a lack of attentional flexibility in classroom activities”.

This is an example scenario that goes along with the findings that children with high intelligence (e.g., gifted children) tend to have a high WM capacity (Aubry et al. 2021) but not necessarily all have good executive functions (Hernández Finch et al. 2014), and that EF contributes to academic success over and above intelligence (Latzman et al. 2010; Moffitt et al. 2011; Zelazo et al. 2016). The present study aims to shed more light on the shared and unique variances in inhibition, shifting (measured with the Hearts and Flowers task; Diamond et al. 2007), working memory, and intelligence in a sample of 6-year-old children. It further examines monitoring and cognitive control as a potential underlying mechanism that might explain the link, through post-error slowing in the EF task, which is a more specific and clearer indicator of metacognitive processes and top-down control (Regev and Meiran 2014).

EF, WM, and intelligence have indeed been conceptualized as strongly related but distinguishable theoretical constructs (Blair 2006). Although there are numerous and differing definitions for each construct, we follow Miyake et al. (2000) in defining EF as a set of distinct higher-order cognitive processes that guide goal-oriented, adaptive, and flexible behavior and the top-down cognitive and behavioral control that is necessary, in particular, in new, complex, and demanding tasks. EF is widely assumed to consist of three main components: inhibition, WM (updating), and shifting (Diamond 2013). WM, also often conceptualized as a component of EFs, is defined as storing and monitoring task-relevant information and replacing this information with more recent task-relevant information when needed. (Fluid) Intelligence is typically similarly defined as a higher-order cognitive ability, allowing complex and abstract reasoning used in novel and challenging situations (Cattell 1971).

Some studies, including ones adopting longitudinal latent variable analyses, find EF to be unitary until around 9 years of age, after which WM appears to be distinguishable from inhibition and shifting (Brydges et al. 2014; Shing et al. 2010). Moreover, a unitary EF was found to predict intelligence at both 7 and 9 years of age (Brydges et al. 2012). Yet, at least one other longitudinal study found that, at both 5 and 6 years of age, inhibition is distinguished as one factor from WM and shifting as another unitary factor (Usai et al. 2014). Moreover, as it will become clearer in the next paragraphs, there is considerable variation in the evidence regarding the existence and the nature of the inter-relations between the components of EF, as well as their relation to intelligence in children younger than 9 years. Because our aim is to shed more light on the intricate relations between these constructs, we use a separate task to assess working memory, inhibition and shifting, and fluid non-verbal intelligence.

Empirical evidence points to close links between these constructs already in childhood. In 7- to 13-year-old children, intelligence is associated with all three components of EF, and mainly with the updating (WM) component (Brydges et al. 2012; Duan et al. 2010; Gómez-Pérez and Calero 2022; Krumm et al. 2018). This is also in line with the findings in young adults that WM, but not inhibition or shifting, is associated with intelligence, even after controlling for the inter-EF correlations (Friedman et al. 2006). Nonetheless, training task-switching (i.e., shifting) improves not only task-switching but also inhibition, verbal and visuospatial WM, and fluid intelligence in children aged 8 to 10 (Karbach and Kray 2009), implying that shifting may also contribute to intelligence and WM. Training inhibition has been found to improve fluid intelligence but not necessarily WM in 4- to 5-year-olds (Thorell et al. 2009; Zhao et al. 2011), implying that inhibition may contribute at least to intelligence, if not WM. It is, however, important to note that the findings of training studies are rather inconsistent and should be taken with caution, also considering the general conclusion that the transfer in EF training appears to be narrow (Diamond and Ling 2016). There are only a few longitudinal studies that investigate the relationships between these constructs across childhood. One study found no longitudinal associations between intelligence at 3 years to parent-reported EF at 4 years (Rahbari and Vaillancourt 2015). Another study found bidirectional longitudinal relations between inhibitory control and intelligence across 4 to 5 years, but not with shifting or WM (Uka et al. 2019).

Studies exclusively focusing on the link between WM and intelligence also find that these abilities develop hand in hand (for a review, see Fry and Hale 2000), and are tightly associated in 4- to 11-year-olds (Cowan et al. 2006; de Abreu et al. 2010; Miller and Vernon 1996; Swanson 2008). Moreover, WM training improves fluid intelligence in children aged 7 to 11 (Klingberg et al. 2005; Zhao et al. 2011). Regarding the visuospatial and verbal WM components, there is very limited evidence on their individual contribution to intelligence. Yet, the scarce existing evidence hints at unique contributions from both (Giofrè et al. 2013; Kuwajima and Sawaguchi 2010; Tillman et al. 2008).

Looking at studies that used the Hearts and Flowers (HF) task as in the current study, a multi-trial EF task commonly used with younger children, intelligence was associated with accuracy in both blocks that, respectively, taxed inhibition and shifting in 4- to 7-year-old children (Blankson and Blair 2016; Romeo et al. 2021). However, WM appeared to relate to accuracy only in the shifting component (Romeo et al. 2021; Traverso et al. 2020). 

Several mechanisms have been put forward to explain the associations between these cognitive abilities. For one, fluid intelligence has been conceptualized to be synonymous with the higher-order reasoning and problem-solving components of EFs, which are supported by all of the lower-order EF constituents, namely, inhibition, shifting, and WM (Diamond 2013). Inhibitory processes, such as suppressing irrelevant information, have been deemed essential for knowledge acquisition and executing many tasks that are defined under intelligent behavior (Dempster 1991). Similarly, inhibiting reflexive impulses allows intelligent behavior, such as rational decision making and functionally adapting to situations (Sternberg 1988; Thurstone 1924). Intelligent behavior is also supported by shifting abilities which allow flexibly switching between different task demands, such as focusing on speed versus accuracy when either demand is more pronounced (Varriale et al. 2021), or solving problems by being able to shift between different perspectives (Rahbari and Vaillancourt 2015). For another, it is argued that WM provides room for holding more information simultaneously in mind to solve problems, which is required in intelligence assessments (Just and Carpenter 1992). Moreover, the cognitive control component of WM, rather than short-term storage, is assumed to contribute to intelligence (Cowan et al. 2006; de Abreu et al. 2010). Cognitive control is often used interchangeably with executive functions and quantified by average accuracy and reaction time (RT) in EF measures (Diamond et al. 2007). It is proposed to be necessary in intelligence measures to evaluate the task, monitor performance, and adapt strategies, and in WM measures to activate the information relevant to the current task and suppress the interference of older or not immediately relevant information (de Abreu et al. 2010). However, as already mentioned above, here, we focus not only on accuracy and RT measures in EF but, additionally, on PES, as a finer quantification of cognitive control. 

Together, the evidence seems to suggest that all EF components are related to intelligence in children, with perhaps WM being somewhat more strongly related. Nevertheless, the empirical evidence in young children is still limited and the underlying mechanisms are unclear. Especially considering that cognitive control may be a critical contributor to intelligence and WM, focusing on further measures in EF tasks that capture control adjustments on a trial-by-trial basis, over and beyond traditional accuracy and RT-based aggregate measures, may prove promising in elucidating the nature of the links between these constructs. One such measure is post-error slowing (PES) and we, therefore, focus on PES in the current study.

### 1.1. PES and Intelligence

Post-error slowing (PES), that is, responding more slowly after committing an error in a relatively simple, multi-trial EF task, is a robust observation in adults (Laming 1979; Rabbitt and Rodgers 1977). PES is typically interpreted as a monitoring process and an adaptive strategy to optimize the accuracy and speed of responding (Botvinick et al. 2001). Namely, to show PES, one is assumed to be monitoring their performance to detect their errors and slowing down in subsequent responding as a control behavior. Whether this potentially adaptive strategy of PES could be linked to other indications of cognitive capacity such as intelligence and WM is an interesting yet vastly understudied question. In children, the dynamics of PES are less well-known. A growing body of research finds evidence for PES in children as young as 3–4 years of age (Jones et al. 2003), and across different executive function tasks (Ger and Roebers 2023; Dubravac et al. 2020). PES, however, appears to be coarser in younger children and becomes fine-tuned with increasing age and experience (Brewer and Smith 1989; Dubravac et al. 2021; Roebers 2022), and is associated with higher accuracy in various EF tasks (Ger and Roebers 2023; de Mooij et al. 2021).

It is surprising that there is only one study in adults, and none in children, that examined the association between intelligence and PES. One study with 6–8-year-olds found no significant associations between neurological markers of error monitoring such as error-related negativity [ERN] and error positivity [Pe] and intelligence (Danovitch et al. 2019). Varriale et al. (2021) looked at the relationship between PES in a Go/NoGo task and IQ in adults and found that PES was predictive of and explained an additional 16% variance in IQ over and above accuracy and RT in the Go/NoGo. However, the association between PES and IQ was negative, indicating that low-ability individuals showed a larger PES compared to high-ability individuals. This was interpreted to suggest that low-ability individuals may have a slower rate of evidence accumulation (based on drift-diffusion modeling; for more information see Ratcliff et al. 2008) or difficulties with setting the optimal response threshold after an error. However, in children, a larger PES might also indicate a non-strategic overreaction to an error because the necessary monitoring and cognitive control processes are not yet fully developed, in line with the age-related changes in PES mentioned above. Therefore, the association between PES and intelligence in young children, if any, might be expected to be positive.

### 1.2. PES and Working Memory (WM)

Given that WM appears to be the EF component that is more robustly related to intelligence than inhibition and shifting in children, it is interesting to examine its relation with further measures of cognitive control in a typical EF task, specifically PES. As WM is especially necessary to update the rules after committing an error, it may be related to post-error slowing as the need to access the rules while at the same time needing to attend to the stimuli in the trial may both contribute to the slowing.

A recent study by McDougle (2022) with adults using an instrumental learning task in which participants were to associate various stimuli with presses on certain keys found that PES decreased with an increasing working memory load (a higher load is imposed by more stimulus-response associations that participants needed to learn). This suggests that WM may play a role in PES and individuals with a higher WM capacity may show a larger PES in an EF task as they would be less susceptible to the WM load of the task. 

To our knowledge, currently, there is no study directly testing WM in relation to PES in children, with the exception of Stins et al. (2005) who found no significant correlations between WM and PES in either a Simon or a Flanker task in 12-year-olds. The authors suspected that the low number of errors in the tasks may have been the reason for the lack of significant correlations.

### 1.3. Current Study

The associations between EF, WM, and intelligence are relatively well-established. Yet, the underlying mechanisms that play a role in the link between these constructs are still unclear. Here, we investigate monitoring and cognitive control as one possible mechanism. The evidence regarding the associations between PES (as an indicator of monitoring and cognitive control in an EF task), WM, and intelligence is very limited, especially in young children. In the current study, we examine these associations in kindergarten children (i.e., ages 5–7) because these ages are critical in the development of EF (Carlson 2005; Davidson et al. 2006). Hence, we study the relationship between EF, WM, and intelligence not only through the accuracy and RT-based measures of EF but also with PES in EF. Based on the limited prior research findings, we expect to find a relationship between PES and intelligence, most probably a positive relationship, but the direction of the relationship is less clear. 

Prior research did not find a significant association between WM and PES in 12-year-olds. However, here, we use a different EF task (i.e., Hearts and Flowers) than the ones employed earlier (i.e., Simon and Flanker tasks) and test a younger age group. In addition, considering the relatively well-established associations between WM and EF as briefly reviewed above, with monitoring and cognitive control as the potential underlying mechanism, which is well-manifested in PES, we expect significant associations between WM and PES in an EF task.

In sum, we expected both intelligence and working memory (both verbal and visuospatial) to be related to the inhibition and shifting components of EF in young children. We also expected both intelligence and working memory (both verbal and visuospatial) to be related to post-error slowing in both blocks of the EF task with no clear but potentially positive direction of these associations. Yet, we expected intelligence to predict post-error slowing over and above working memory.

## 2. Method

### 2.1. Participants

The data come from the pre-testing of 174 kindergarten children as part of a larger intervention project. Children were between the ages of 5.5 and 7.5. Children who committed more than 40% errors in any of the blocks of the HF task (*N* = 23) were excluded from the analyses following Ger and Roebers (2023), to ensure that children understood the task well and performed above chance. Four more children were excluded due to not completing the dwarf task. The mean age of the final sample included in the analyses (*N* = 147, 47% female) was 6.5 years (*SD* = 0.3). An a priori power analysis to obtain 90% power for an average effect size of r = 0.30 derived from the reviewed literature for a Pearson’s correlation analysis estimated a required sample size of 111, ensuring that our final sample was sufficiently powered. Children came from urban and rural areas of central Switzerland and mainly from families of lower- to upper-middle class (see Table 1 for more detailed sample characteristics). They were recruited by contacting interested kindergarten teachers. Parents of participating children gave written informed consent. The study was approved by the local ethics committee and was conducted in accordance with the Declaration of Helsinki.

### 2.2. Tasks, Materials, and Procedure

All tasks were administered on tablet computers (Samsung Galaxy Tab S4 and Samsung Galaxy Tab A7). Responses were registered with millisecond accuracy through external buttons in the HF task, and by a finger tap on the touchscreen in the remaining tasks. Children solved the tasks individually on the tablet in small groups in a quiet room in their kindergarten. The tasks were solved as the pre-test of a larger training study. The pre-test was conducted on two separate days with a maximum of 2 days in between. In the first session, children were first tested on the Hearts and Flowers task followed by the Mole task (visuospatial WM) and then the Odd-Item-Out subset of the Reynolds Intellectual Assessment Scale (RIAS). In the second session, children were first tested on a paired associate task (not examined and included in this study) followed by the Dwarf task (verbal WM). 

### 2.3. EF: Hearts and Flowers (HF) Task

The Hearts and Flowers (HF) task, adapted from Diamond et al. (2007), was used to assess executive functions. This task was chosen because it is a multi-trial task that allows calculating post-error slowing. Moreover, it is used worldwide with young children to assess EF, it provides sufficient variance to explain EF development from 4 to 26 years (Davidson et al. 2006), has high reliability (Rosas et al. 2019), and shows good concurrent validity with other EF tasks such as Stroop-like tasks (Brocki and Tillman 2014). The task is composed of three blocks presented in the following fixed order: hearts, flowers, and mixed. In the hearts (congruent) block, a heart appears on the left or right of the screen in each trial and children have to press a button on the corresponding side. This block consists of 24 trials and establishes a prepotent response. In the flowers (incongruent) block, a flower appears on the left or right of the screen in each trial and children have to press the button on the opposite side. This block consists of 36 trials and requires inhibiting the previously established prepotent response. In the mixed block, heart and flower trials are presented in a pseudo-randomized order, in which a heart trial always surrounds a flower trial. The mixed block consists of 48 heart (congruent) and 12 flower (incongruent) trials and requires rule switching. 

Before the test trials, children always receive instructions for what they need to do and that they should answer as fast as possible but also slow enough to answer correctly. Children also participate in four practice trials before test trials in each block. The stimuli were presented for 600 ms. Trials lasted until the child’s button press and the inter-trial interval was 500 ms during which a fixation cross appeared on the screen. The accuracy and reaction time (RT) of the response in each trial were measured. Because the trials proceed once the child gives an answer, extremely long trials are possible in case of off-task periods due to inattention or being distracted. For this reason, we removed trials with a reaction time longer than 2500 ms following the maximum trial length of Wright and Diamond (2014) and those with a reaction time shorter than 250 ms as they are too short to have been executed as a response to the current stimulus.

Regarding the indices in EF, we calculated accuracy as the percentage of correct answers in each of the HF incongruent and mixed blocks; the congruency effect as the mean RT in the incongruent block minus the mean RT in the congruent block (i.e., the larger this value, the longer the time taken to inhibit a prepotent response); and the shift cost as the mean RT in the mixed block minus the mean RT in the incongruent block (i.e., the larger this value, the longer the time taken to shift between rules). Accuracy in the incongruent block and the congruency effect served as indicators of inhibition. Accuracy in the mixed block and shift cost served as indicators of shifting. As the index of monitoring and cognitive control, we calculated PES by the traditional method of calculating the mean individual RT of correct post-error trials minus the mean individual RT of correct post-correct trials (Dutilh et al. 2012).

### 2.4. Verbal WM: Dwarf

This is a backward color recall task adapted from Zoelch et al. (2005). This task was chosen because it is valid and has acceptable retest reliability (Schmid et al. 2008), it represents a standard verbal span task but does not require digit knowledge and is nicely embedded in a child-appropriate cover story. In the cover story, a dwarf walking through the woods has a sack full of colorful frisbees. However, the sack has a hole and frisbees fall out of the sack. The task of the child is to watch the color of the frisbees that fall out and help the dwarf to collect them in the reverse order. Namely, circles of different colors appeared in the middle of the screen for 1000 ms replacing each other with an interstimulus interval of 500 ms. Afterwards, a palette of 6 colors was presented on the screen, from which the child needed to select the color of the frisbees in the reverse order than presented. The number of frisbees (i.e., span) starts at 2 and increases step by step up to 7 as long as the child correctly answers at least half of the total number of trials in a given span (i.e., 3 trials out of 6 total trials on each span). Hence, the task has a stopping rule of at least 50% performance within a span. Children first receive an example for 2-span, then 3 practice trials for the 2-span, then the test trials of the 2-span. Later, if they pass the 2-span stage, they receive an example for the 3-span followed by 3 practice trials and the test for the 3-span. Afterwards, no further examples and practice trials are given but only the test trials. If children fail the practice trials, they receive feedback and additional instruction from the experimenter. The test trials do not start until after the child passes all 3 practice trials on the 2-span task.

As the index of verbal WM, we calculated accuracy by taking the sum score of correct trials. A trial is scored as correct when the child reproduces the correct sequence of colors. As there are 6 blocks with 6 trials in each block, scores may range from 0 to 36.

### 2.5. Visuospatial WM: Mole

This is a forward position span task adapted from Frick and Möhring (2016), based on the Corsi Block-Tapping Task (Corsi 1972). This task was chosen because it represents a standard spatial span task but is nicely embedded in a child-appropriate cover story. In the cover story, a mole appears at different locations in a 4 × 4 grid. The task of the child is to memorize the locations where the mole appears and reproduce this sequence in the same order. The mole appears in each field for 1200 ms and children are asked to respond 1000 ms after the last mole disappears from the screen. The inter-trial interval is 500 ms where the empty grid stays on the screen. The items appear in a fixed pseudo-randomized order. The number of locations (i.e., span) starts at 2 and increases step by step up to 7 as long as the child correctly answers at least half of the total number of trials in a given span (i.e., 3 trials out of 6 total trials on each span). Hence, the task has a stopping rule of at least 50% performance within a span.

As the index of visuospatial WM, we calculated accuracy by taking the sum score of correct trials. A trial is scored as correct when the child reproduces the correct sequence of positions. As there are 6 blocks with 6 trials in each block, scores may range from 0 to 36.

### 2.6. Intelligence: Odd-Item-Out Subtest of the Reynolds Intellectual Assessment Scale (RIAS)

Fluid intelligence (i.e., nonverbal IQ) was assessed using the Odd-Item-Out subtest of the Reynolds Intellectual Assessment Scale (RIAS; Reynolds and Kamphaus 2003, German adaptation: Hagmann-von Arx and Grob 2014) adapted to be used on a tablet computer. RIAS scale was chosen due to its good psychometric properties (Andrews 2007) and easiness to computerize it. Children’s task is to find the picture that does not fit in a set of 5–7 pictures in each item based on different and changing features (color, shape, orientation, category, etc.). With this subtest, non-verbal skills such as spatial ability and visual imagery are measured. Children first receive instructions and participate in three practice trials where they receive feedback if they answer incorrectly before they move on to the test trials.

As the intelligence index, we calculated a sum score of the correct answers. Correct answers received one point if answered within 50 s, and 2 points if answered within 30 s. As there are 51 items in total, scores may range from 0 to 102. 

## 3. Results

The pre-registration, anonymized data, and analysis script can be found at: https://osf.io/mg5bj/. The data were analyzed using R [version 4.1.3] (R Core Team 2020).

**Figure 1:**
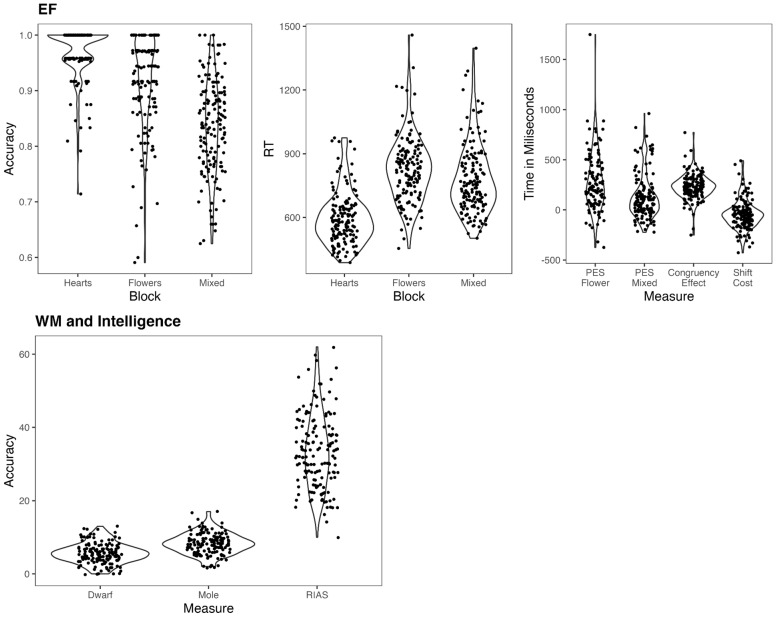
Violin plots of measures from EF, WM, and intelligence tasks.

Descriptive statistics of the indices in each task are presented in Table 2 and visualized in Figure 1. Preliminary directional one-sample Wilcoxon signed-rank tests found that post-error slowing in both the incongruent (*p* < .0001, effect size r = 0.77) and mixed blocks of HF (*p* < .0001, effect size r = 0.52) and the congruency effect in HF (*p* < .0001, effect size r = 0.85) were significantly above 0 but the shift cost in HF was not (*p* = 1). 

In the following, we report our pre-registered and exploratory analyses. We ran Spearman correlations where the normality assumption was violated. Otherwise, Pearson correlations are reported.

**Figure 2:**
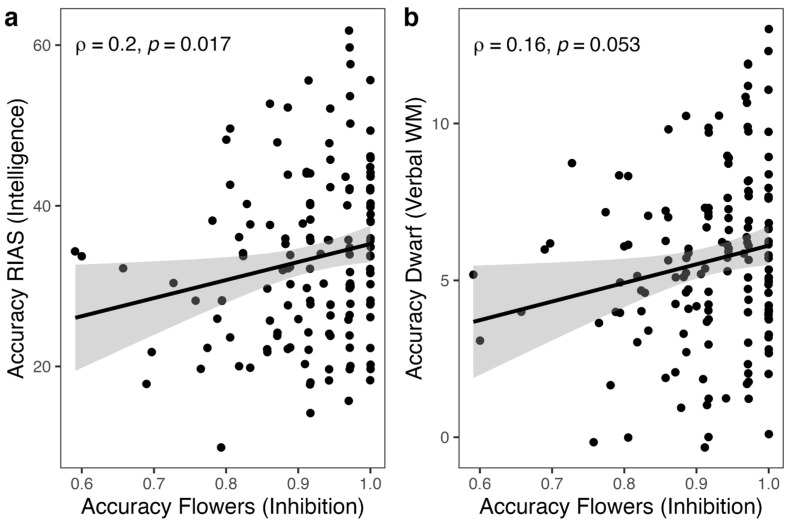
Correlation of accuracy in inhibition with intelligence (**a**) and verbal WM (**b**).

**Figure 3:**
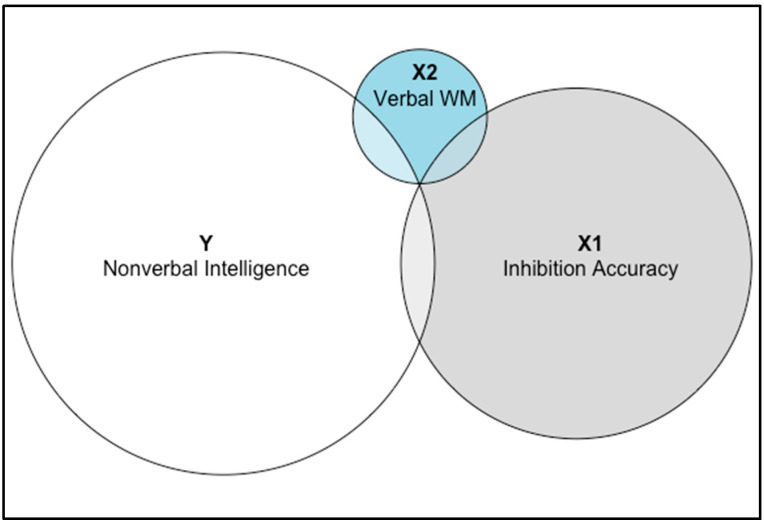
Shared and unique variances between intelligence, verbal WM, and inhibition accuracy.

Regarding inhibition, accuracy in the incongruent block of HF was positively and significantly correlated with fluid intelligence (*ρ* = 0.20, *p* = .017; Figure 2a) even after controlling for RT (*ρ* = 0.19, *p* = .019). Accuracy in the incongruent HF was also marginally positively correlated with verbal WM (*ρ* = 0.16, *p* = .053; Figure 2b), which turned significant when controlling for RT (*ρ* = 0.16, *p* = .049), but not with visuospatial WM (*ρ* = −0.01, *p* = .954). Figure 3 shows a Venn diagram generated using the R package ‘eulerr’ (Micallef and Rodgers 2014) to illustrate the extent of shared and unique variances through linear regression model fits. The congruency effect (i.e., time taken to inhibit the prepotent rule) was not associated with intelligence or WM. Regarding shifting, although accuracy in the incongruent and mixed blocks were positively correlated (*ρ* = 0.33, *p* < .0001), neither accuracy in the mixed block of HF nor the shift cost (i.e., time taken to shift between rules) was associated with either intelligence or WM (all *p*s > .181). Regarding cognitive control, PES in either block of EF was not associated with intelligence or WM (all *p*s > .056), even after controlling for age. Finally, verbal WM and visuospatial WM were positively correlated (*r* = 0.20, *p* = .015), while intelligence was positively correlated only with verbal WM (*r* = 0.16, *p* = .049). 

Further exploratory partial correlation analyses showed that the correlation between inhibition accuracy and intelligence held when controlling for verbal WM (*ρ* = 0.18, *p* = .034), whereas the correlation between verbal WM and intelligence was no longer significant when controlling for inhibition accuracy (*ρ* = 0.13, *p* = .136).

Despite the lack of significant correlations, we went on to run our two pre-registered hierarchical linear regression analyses to predict PES, respectively, in the incongruent and mixed block of the EF task by WM and intelligence. In the first step, we entered the accuracy and RT from the HF task as control variables. We entered the visuospatial and verbal WM in the second step and intelligence in the final step. The results are presented in Table 3.

We exploratorily investigated whether the trajectory of PES, namely, the magnitude of post-error slowing (i.e., how extremely children slow down after an error) throughout the course of a block would change as a function of intelligence, which we may have missed in the overall PES that we looked at. To this end, we ran a mixed linear regression, using the R package ‘lmerTest’ (Kuznetsova et al. 2017), with RT as the dependent variable; intelligence, the block, and the order of the error within the block (i.e., whether it is the first error, second error, third error, and so on), and their three-way interaction as fixed effects; the overall accuracy as a control variable; and the participant as random effects. We calculated the PES for each error by subtracting the average RT of the post-correct correct trials within the block from the RT of that error. Model comparisons revealed neither a significant three-way interaction (*Χ*^2^(3) = 0.46, *p* = .927) nor a two-way interaction between the block and index (*Χ*^2^(3) = 0.48, *p* = .487). However, there was a main effect of the block (*F*(1, 1094) = 22.07, *p* < .0001) and index (*F*(1, 1034) = 16.29, *p* < .0001), whereby the magnitude of PES was lower in the mixed block than in the flowers block (mean difference = 126.4 ms), and PES reduced throughout the course of a block (by 17.6 ms with every subsequent error), independent of the block and children’s intelligence.

In parallel, we explored the trajectory of the response accuracy throughout the course of a block to see whether it overlaps with the course of PES. We used a similar analytical approach as above, with the difference being that we used a generalized linear mixed model (GLMM) instead of a linear mixed model, where the outcome variable was the binary-coded correctness of a response. Because the models with the order of the trial (i.e., trial number) as a fixed effect did not converge, we instead chunked every six trials and used this chunk number in the model. We found that, with each proceeding trial, the probability of giving a correct response decreased in the mixed block by 1% (*p* < .0001)^1^, while it did not significantly change in the flowers block despite showing an increasing trend of 0.8% (*p* = .11).

Given that in the previous literature, the direction of the links between the constructs is unclear for the RT measures of EF, we explored these links in the current study. The mean RT in both the incongruent and mixed block of HF was negatively correlated with visuospatial WM (r = −0.29, −0.21, *p* < .0001, .01, respectively), even after controlling for accuracy in each block. The mean RT in the mixed block of HF was positively correlated with intelligence (*r* = 0.23, *p* < .01), even after controlling for accuracy in the mixed HF. Note that non-parametric correlations yielded the same pattern of results.

We also explored the links between WM and EF by looking further into other measures from the WM tasks. Specifically, we examined the span score (i.e., the highest span where the child scored at least 50%) and the total number of correct locations in the visuospatial WM task (regardless of the correct order), and the span score and the total number of correct colors in the verbal WM task (regardless of the correct order). Accuracy in the incongruent block of HF was positively correlated with the correct colors in the verbal WM task (*ρ* = 0.24, *p* < .01). The PES in the mixed block of HF was positively correlated with the span score in the verbal WM task (*ρ* = 0.19, *p* = .025). The congruency effect in HF was negatively correlated with the correct location in the visuospatial WM task (*r* = −0.16, *p* = .049). No other correlation reached significance.

## 4. Discussion

In this study, we examined the associations between numerous measures of executive functions, post-error slowing as a manifestation of metacognitive processes, visuospatial and verbal working memory, and intelligence in kindergarten (5.5- to 7.5-year-old) children. Partly in line with our expectation, we found the accuracy in the inhibition component of EF to be positively associated with intelligence and verbal WM, and RT thereof to be negatively related to visuospatial WM. Only the RT, but not accuracy, in the shifting component of EF was positively related to intelligence and negatively related to visuospatial WM. In contrast to our expectation, neither the congruency effect, shift cost, nor PES in EF was associated with intelligence or WM. Verbal and visuospatial WM was associated with each other but intelligence was only associated with verbal but not visuospatial WM. 

### 4.1. Executive Functions (EF), Working Memory (WM), and Intelligence

Our finding that only the accuracy in the incongruent block (taxing inhibition) but not in the mixed block of HF (taxing shifting) was associated with intelligence is consistent with one previous study (Duan et al. 2010), and partly consistent with some, which found associations in both blocks in 4- to 7-year-old children (Romeo et al. 2021), and which found an association in the mixed block of HF in 5-year-olds but did not analyze the incongruent block (Blankson and Blair 2016; Traverso et al. 2020). This suggests that around these ages, perhaps inhibition is more robustly linked to intelligence than shifting (Uka et al. 2019). An interesting study by Ren et al. (2017) showed that, in adults, WM and shifting were associated with the component of intelligence reflected in the learning and use of a strategy/type of solution. In contrast, inhibition was associated with the component of intelligence reflected in inhibiting task-irrelevant information and remaining on task (Ren et al. 2017). Our findings could similarly imply such a mechanism in young children whereby their inhibition was the more influential component of their performance in the intelligence test through a better focus on the task, rather than the ease with which a child comes up with solution strategies per se.

Corroborating the seemingly more critical role of inhibition in intelligence, we found accuracy in inhibition to be correlated with intelligence even after partialling out the contribution of verbal WM, but the correlation between verbal WM and intelligence was no longer significant after partialling out the contribution of inhibition accuracy. This finding further suggests that inhibition may be necessary over and above WM in intelligence measures in 6-year-olds. Although, at first glance, it seems that this finding is not compatible with the body of research nominating working memory as the most strongly related to intelligence among the three main EF components (e.g., Duan et al. 2010), it should be noted that this work mostly focused on children older than 6 years of age. Inhibition is known to be the earliest developing EF component (Best and Miller 2010), and, to our knowledge, it is the EF component that is shown to improve fluid intelligence when trained with the youngest children, specifically ages 4 to 5 (Thorell et al. 2009; Zhao et al. 2011). Therefore, inhibition could be more likely to mark the earliest links to more general cognitive abilities such as intelligence. 

Our exploratory analyses revealed that the mean RT in shifting was positively associated with intelligence, even after controlling for accuracy. Namely, children with higher intelligence scores took longer to respond in the block of the EF task that requires shifting between different task rules. This is counterintuitive as one might expect more intelligent individuals to be faster in responding, due to faster information processing (Kail 2000; Sheppard and Vernon 2008), without having to trade it off for higher accuracy. Given that the individuals’ accuracy in the incongruent and mixed blocks were also highly positively correlated, and the former was associated with intelligence, it is possible that inhibition is also responsible for the slower responses of more intelligent children in the shifting block. Namely, more intelligent children can inhibit themselves better to respond more slowly in a block that requires them to shift between rules. Alternatively, more intelligent children’s slower responding in the mixed block compared to their less intelligent counterparts could be a reflection of a more advanced strategy, whereby they are more aware of the higher demands of this block and choose to respond slower. 

The finding that, in the inhibition block, accuracy was related to verbal WM and RT was related to visuospatial WM could be explained by the nature of the working memory demand in this block. That is, children might have needed to hold the rule that they need to press on the opposite side where a flower appears on the screen in their verbal WM to press on the correct side. In contrast, they might have needed to rely on their visuospatial WM to remember where the stimulus had appeared on the screen and avoid the interference from the previous stimulus location to respond quickly. The association between the RT in the shifting block and visuospatial WM could be explained with the same reasoning, although the lack of a relationship between the accuracy in shifting and verbal WM needs further elucidation.

It is surprising that our measure of intelligence was positively associated with verbal WM, but not visuospatial WM, especially considering the nonverbal and visuospatial ability-based nature of our intelligence measurement. This finding is still in line with previous work suggesting that WM is to be robustly associated with intelligence (Duan et al. 2010; Johann and Karbach 2022). However, it contradicts the previous research that found both visuospatial and verbal components of WM contribute to intelligence, measured with Raven’s Progressive Matrices, a nonverbal measure similar to ours (Tillman et al. 2008).

The shift cost in EF was not associated with intelligence or WM. Still, our exploratory analyses revealed the congruency effect in EF to be negatively correlated with the correct location in the visuospatial WM task. This indicates that children who slow down less in the face of incongruence relative to their baseline speed are better at remembering the locations, albeit not in the correct order, in a position span task. Again, in parallel to our other findings, the inhibition, but not shifting component, appears to be related to WM whereby better inhibitors remember the visuospatial locations better. However, it should also be noted that the shift cost was not significantly different than zero; namely, children did not have to slow down their responses in the mixed block of the EF task where they had to shift between rules relative to the incongruent block where they had to inhibit the prepotent response. This pattern is in line with Roebers (2022) who found no shift cost in 6-year-olds, but in 7-year-olds, and even greater in 8-year-olds, indicating that, with increasing age, children adapted their cognitive control better to the increasing task demands. Hence, the shift cost may differentiate based on intelligence or WM only at a later age, around 7 years, when it emerges as a manifestation of cognitive control. 

### 4.2. PES in Relation to Intelligence and WM

As an indicator of metacognitive processes such as monitoring one’s accuracy, detecting errors, and taking actions to avoid further errors, we focused on post-error slowing (PES) within EF measures. One recent study with adults found PES in EF and intelligence to be linked (Varriale et al. 2021) and a few studies hinted at indirect links (Hirsh and Inzlicht 2010; Moreno et al. 2011). The lack of such an association in our study, including the course of PES throughout a task block, suggests that this association might emerge only at a later age. Nevertheless, our descriptive and exploratory findings replicate recent previous findings that PES is robustly observed in young children (Ger and Roebers 2023). We further show that children reduce their PES throughout the course of a block, which may be a strategic down-regulation of the magnitude of slowing. Therefore, although kindergarten children display monitoring and cognitive control in a multi-trial EF task, this appears to be a strategy that is not differentiating among individuals as a function of their intelligence, unlike adults. Regarding WM, our exploratory analyses revealed a positive correlation between PES in the mixed block of HF and the span score in the verbal WM task. Although PES did not correlate with our hypothesized accuracy score in the verbal WM task, the positive correlation with the span score may still be an indicator of an emerging relationship. To our knowledge, only one previous study (with 12-year-old children) examined the relationship between PES and WM and found no significant associations (Stins et al. 2008). Together with this and our null findings in 6-year-olds, we refrain from overinterpreting this positive relationship further.

Examining 6- to 12-year-old children, PES is observed to be more exaggerated in younger children compared to older children and adults (Dubravac et al. 2020, 2021; Roebers 2022). That is, younger children slow down more extremely after an error and, in developmental time, become better at fine-tuning their slowing to an optimal magnitude, just enough to be more accurate in the subsequent trials. Thus, at our sample’s age range (i.e., 5.5–7.5 years), children may still be in the early phases of developing post-error slowing as a metacognitive strategy. They may therefore not yet show individual differences in how optimally they employ post-error slowing, which could potentially relate to their other cognitive skills such as WM or intelligence.

Nevertheless, our exploratory analyses showed that children reduced the extent of their slowing throughout the course of a block in both the incongruent and mixed blocks of the EF task, independent of their intelligence. Interestingly, again independent of intelligence, the probability of giving a correct response did not change in the incongruent block while it decreased in the mixed block as a function of trial number. That is, throughout the course of a block, the overall pattern of decreasing PES overlapped with an overall pattern of stable accuracy in the incongruent block but with decreasing accuracy in the mixed block, although children start with a similarly high probability at the beginning in both blocks. This raises the possibility that, in the relatively less demanding incongruent block, children may come to realize, as they progress with the trials, that slowing down after an error may not be that necessary and may even interfere with their subsequent performance. In contrast, in the relatively more demanding and also longer mixed block, increasing fatigue toward the end of the block may be a common cause of both decreasing accuracy and PES. Potentially, in the face of high demands, the reduced exertion of cognitive control, reflected in reduced PES, may lead to a reduced probability of correct responses. This would also align with the findings of Ger and Roebers (2023) that PES may be a strategy that works to obtain a high accuracy only in sufficiently demanding EF tasks, such as the mixed block of the HF task. In sum, 6-year-old children appear to show post-error slowing, in a seemingly strategic manner to a certain extent, yet predictable individual differences in post-error slowing may develop later in developmental progression.

We focused on monitoring within the context of a multi-trial executive functioning task where participants are to respond under time pressure. We assessed PES as an indicator that participants track their performance (accuracy and speed of responding), detect their errors, and slow down after errors as a control of future performance. We reasoned that WM may relate to PES because taking time to update the rules becomes especially important after committing an error. In addition, more intelligent children may be better in evaluating their performance. Metacognitive monitoring has mainly been researched in the metacognition literature with more explicit measures such as reporting confidence judgments about the accuracy of one’s responses, usually without time constraints. In this context, WM is assumed to support keeping information in mind while giving confidence judgments. In addition, as with error monitoring, more intelligent children may be better at evaluating their self-performance in metacognitive tasks. Consistent with our findings, some previous research documented a lack of significant associations between metacognitive monitoring and WM in children at ages 4 to 8 (Bryce et al. 2015; Kälin and Roebers 2020, 2022; Roebers et al. 2009) and that gifted children did not necessarily display better metacognitive monitoring before school age (Carr et al. 1996; Snyder et al. 2011). Considered together with the overlap with the metacognition literature, monitoring may thus be expected to be associated with other cognitive constructs such as WM and intelligence only in school age.

Other possible explanations concerning the lack of associations with PES could stem from the complexity of the constructs at hand, conceptualization problems, or measurement issues (see Baggetta and Alexander 2016, for a systematic review on EF). There has long been a debate on defining EF, WM, and intelligence, and several theories have been put forward to conceptualize these complex constructs (Uka et al. 2019). Correlational, neuroimaging, and developmental evidence point to a lack of a complete overlap or dissociation (Diamond 2013; Miyake et al. 2000). Regarding measurements, the task impurity problem is commonly addressed in the cognitive literature (Miyake et al. 2000). For instance, a task designed to measure the cognitive flexibility component of EF is likely to also tap inhibition and working memory components. Moreover, PES is indexed by reaction times while the other examined constructs rely mainly on accuracy.

### 4.3. Limitations and Future Directions

The current study used a single performance-based task to assess each of the skills at hand. Previous research has shown that there may be differences in the assessment of cognitive skills depending on the source of the assessment. For instance, performance-based assessments of executive functions, which may lack in ecological validity, do not always correlate with parent or teacher evaluations (Isquith et al. 2005). Therefore, using a more varied battery of tasks and varying sources could reduce measurement error and alleviate the task impurity problem mentioned before, which might have contributed to the current nonsignificant results. Moreover, all our tasks were computerized, and children were tested individually but still together with other peers in small groups. These testing characteristics might also have contributed to the pattern of findings; for instance, there might have been more room for distraction by peers. Future studies replicating the current analyses in a setting where children are tested alone in a room and/or with pen-and-paper tasks are needed to warrant the external validity of the current findings. Finally, intellectual abilities including fluid intelligence, WM, EF, and performance monitoring and control continue to change in essentially the whole lifetime and differ in their structural organization across age (Hämmerer et al. 2014; Li et al. 2004). Therefore, testing the inter-relations between these abilities in different age groups, ideally in a longitudinal design, is a crucial future direction to capture a more comprehensive understanding of the questions at hand.

## 5. Conclusions

The association between the inhibition component of EF, WM, and intelligence, and the lack of associations with shifting or PES in EF suggest that EF, WM, and intelligence might be linked through inhibition rather than monitoring and cognitive control, at least at kindergarten ages. Monitoring and cognitive control continue to develop further throughout childhood, and it may explain some of the shared variances between these three constructs only once it has reached a substantial level of variance and optimization and manifested in measures such as PES or the shift cost. A promising future direction is to longitudinally assess WM and intelligence in children and capture the age at which significant associations with indications of their cognitive control such as post-error slowing in EF may emerge. One practical implication of the current findings is that it may be promising to target training inhibition as a potential shared component between EF, WM, and intelligence to have an influence on all three constructs. This may be particularly important to reduce costs and maximize benefits when faced with intervention-related limitations.

## Figures and Tables

**Table 1:** Sample characteristics.

Variable			Nationality		
Mother			Father		
	*N*	%		*N*	%
Swiss German	107	76	Swiss German	99	70
Turkish	5	3.6	Turkish	6	4.3
German	4	2.8	German	4	2.8
Macedonian	4	2.8	Italian	6	4.3
Other	21	15	Kosovan	4	2.8
			Other	22	16
			**Child Language**		
**First Language**			**Second Language**		
	***N***	**%**		***N***	**%**
German	56	40	German	19	72
Swiss German	49	35	Italian	8	5.7
Turkish	8	5.7	Other	13	9.2
Albanian	6	4.3	None	101	72
Other	22	16			

*Note.* Demographics data could not be obtained from 6 children; hence, the descriptives are based on *N* = 141. Of the children, 2.8% heard English and 2.1% heard another language as third language.

**Table 2:** Descriptive statistics of measures from EF, WM, and intelligence tasks.

Measure	Min	Max	Mean	SD
	HF			
Accuracy Hearts	71%	100%	97%	5%
RT Hearts	388	975	593	122
Accuracy Flowers	59%	100%	92%	8%
RT Flowers	454	1459	825	154
PES Flowers	−374	1748	282	290
Accuracy Mixed	63%	100%	84%	8%
RT Mixed	502	1397	779	162
PES Mixed	−222	961	122	215
Congruency Effect	−251	771	232	123
Shift cost	−427	490	−46	145
	WM			
Accuracy Dwarf	0	13	6	3
Accuracy Mole	2	17	8	3
	Intelligence			
Accuracy RIAS	10	62	33	10

*Note*. All RTs, congruency effect, and shift cost are in the unit of milliseconds. The maximum score in Dwarf and Mole is 36, and in RIAS is 102.

**Table 3:** Results of the hierarchical regressions predicting PES in the incongruent and mixed HF.

	Incongruent HF				Mixed HF			
Predictor	B	SE B	Adj. R^2^	ΔR^2^	B	SE B	Adj. R^2^	ΔR^2^
Step 1			−0.009	−0.009			0.134 ***	0.134 ***
Accuracy	0.09	0.10			0.20 *	0.08		
RT	−0.04	0.10			0.27 **	0.08		
Step 2			−0.014	−0.005			0.124 ***	−0.010
Verbal WM	0.08	0.10			0.01	0.08		
Visuospatial WM	−0.09	0.10			0.05	0.08		
Step 3			−0.023	−0.009			0.118 ***	−0.006
Intelligence	−0.00	0.01			−0.00	0.01		

*Note*. * *p* < .05, ** *p* < .01, and *** *p* < .001. *B* represents standardized beta coefficients.

## Data Availability

The anonymized data are publicly available at: https://osf.io/mg5bj/ (DOI: 10.17605/OSF.IO/MG5BJ).

## Note

1 Note that this is the linear equivalent of the original estimate of 6% for every six trials.
